# Preparation of Co-Amorphous Systems by Freeze-Drying

**DOI:** 10.3390/pharmaceutics12100941

**Published:** 2020-09-30

**Authors:** Melvin Wostry, Hanna Plappert, Holger Grohganz

**Affiliations:** Department of Pharmacy, University of Copenhagen, Universitetsparken 2, 2100 Copenhagen, Denmark; melvin.wostry@gmail.com (M.W.); Hanna.Plappert@uni-duesseldorf.de (H.P.)

**Keywords:** co-amorphous, freeze-drying, surfactant

## Abstract

Freeze-drying was evaluated as a production technique for co-amorphous systems of a poorly water-soluble drug. Naproxen was freeze-dried together with arginine and lysine as co-former. To increase the solubility of naproxen in the starting solution, the applicability of five surfactants was investigated, namely sodium dodecyl sulfate, pluronic F-127, polyoxyethylene (40) stearate, tween 20 and TPGS 1000. The influence of the surfactant type, surfactant concentration and total solid content to be freeze-dried on the solid state of the sample was investigated. X-ray powder diffraction and differential scanning calorimetry showed that the majority of systems formed co-amorphous one-phase systems. However, at higher surfactant concentrations, and depending on the surfactant type, surfactant reflections were observed in the XRPD analysis upon production. Crystallization of both naproxen and amino acid occurred from some combinations under storage. In conclusion, freeze-drying was shown to be a feasible technique for the production of a selection of co-amorphous drug–amino acid formulations.

## 1. Introduction

Co-amorphous systems have gained interest as a promising system to stabilize poorly water-soluble drugs in an amorphous form over the last few years [1]. These co-amorphous systems are defined as mixtures between the active pharmaceutical ingredient (API) and one or more small molecular compounds, in order to differentiate them from polymeric glass solutions [2]. Although drug–drug combinations were originally the focus [3,4], this has changed as drug–excipient combinations result in more universally applicable systems. Amongst the investigated excipients, amino acids (AA) have to date been given the most attention [5,6], although other classes such as organic acids [7,8], saccharine [9,10] and nicotinamide [11,12] also were investigated.

The majority of articles investigating co-amorphous systems used vibrational ball milling as a production technique [13]. However, this technique is more suited to laboratory scale production and is of limited interest for industrial scale production. Thus, recently, more focus has been placed on scalable techniques, especially spray-drying [14,15]. It has been shown that spray-drying is a viable technique for the production of co-amorphous systems, frequently even surpassing the results obtained via ball milling. This is because ball milling requires a mechanical breakdown of the existing crystalline structures, whilst spray-drying is based on a molecularly dispersed system, namely a solution. On the downside, spray-drying might not be applicable for systems with low glass transition temperatures of the product, as a sticky product would be obtained if the glass transition temperature was below the outlet temperature of the process. In addition, the rather frequent requirement to use organic solvents can be viewed as a disadvantage, considering both patients and the environment [16]. It would thus be of interest to investigate whether another technique that can yield amorphous products can be applicable for the production of co-amorphous systems, despite the financial costs usually associated with this method: freeze-drying.

From a practical perspective, freeze-drying is not a bulk technique in the usual sense, where upscaling is a major challenge, as the primary container (the vial) is the same on the laboratory and industrial scale. First and foremost, the process will need to be adapted from laboratory to production scale. Process optimization of freeze-drying cycles is a field of research with numerous articles and is outside of the scope of this manuscript but the interested reader is referred to a quality by design approach towards the upscaling of freeze-drying [17]. Freeze-drying in general is considered as a gentle drying method; however, mostly, its applications are directed towards proteins [18] and other macromolecules [19]. Freeze-drying is well-known as a method to produce amorphous material and, although freeze-drying of solutions of smaller molecules is frequently performed, very little literature can be found for the intentional production of co-amorphous substances [20,21].

With regard to freeze-drying, an obvious challenge comes to mind when considering the technique for co-amorphous systems. A co-amorphous system is usually considered for poorly water-soluble substances, whilst freeze-drying is most frequently performed from an aqueous solution. To obtain an amorphous system, the API and the co-former should be dissolved in the medium and therefore measures have to be undertaken to enable this. In order to address this issue, several modes of action come to mind: (1) the application of a solvent mix of water and organic solvent that dissolves both API and co-former, (2) pH adjustment for ionizable substances and (3) the use of surfactants. With regard to (1), only a limited number of organic solvents are relevant for freeze-drying as the majority of organic solvents have melting points which are too low to be of relevance. Frequently applied organic solvents are tert-butanol and dimethyl sulfoxide [22,23,24]. With regard to (2), the use of buffers should be considered carefully as strong pH changes can occur due to selective freezing of the buffer salts. Considering these challenges, it was decided to initiate the investigation with the application of surfactants [25].

The influence of different production techniques (ball milling, liquid assisted grinding and spray-drying) on the properties, including in vivo data, of a system consisting of naproxen and arginine was recently studied in detail [26]. Due to the availability of this reference system, it was decided to perform a detailed investigation based on the acidic BCS class II naproxen (NAP) as model drug, in combination with two basic amino acids, arginine (ARG) and lysine (LYS). In addition, the effects of the five surfactants, sodium dodecyl sulfate (SDS), pluronic F-127 (PF127), polyoxyethylene (40) stearate (P40S), tween 20 (T20) and TPGS 1000 (TPGS), were investigated.

It was the aim of this study to investigate whether freeze-drying could be a viable technique for the production of co-amorphous drug–amino acid systems and to obtain an initial overview of the influence of formulation parameters and surfactants on the product characteristics.

## 2. Materials and Methods

### 2.1. Materials

The acidic drug *(S)*-Naproxen (M_w_ 230,26 g/mol, pk_a_ = 4.15, solubility in water 15.9 mg/L) was purchased from Fagron (Barsbüttel, Germany) and the basic amino acids L-arginine (M_W_ = 174.2 g/mol, pk_a_ = 9.04 and 12.48) and L-lysine (M_W_ = 146.2 g/mol, pk_a_ = 8.95 and 10.53) were purchased from Sigma Aldrich (St. Louis, MO, USA), respectively. The surfactants sodium dodecyl sulfate (M_W_ ∼ 288 g/mol), pluronic F-127 (M_W_ ∼ 12,600 g/mol) and polyoxyethylene (40) stearate (M_W_ ∼ 328.5 g/mol) were also purchased from Sigma Aldrich (St. Louis, MO, USA). Tween 20 (M_W_ ∼ 1227 g/mol) was donated by Merck (Darmstadt, Germany), while the TPGS 1000 (M_W_ ∼ 1513 g/mol) was bought from Isochem (Vert-le-Petit, France). All substances were used as received. All surfactants are considered non-toxic in the used amount. The toxicity was considered based on Pub Chem, the National Library of Medicine, with the following LD 50 values: SDS (rat, oral: 1288 mg/kg), tween 20 (rat, oral: 37 g/kg), pluronic (rat, oral: 22.4 g/kg) and polyoxyethylene stearate (mouse, i.p. 200 mg/kg). No toxicity data were available for TPGS.

### 2.2. Solubility Investigations

An initial screening for promising API–excipient combinations was performed. The success criteria in this screening were based on the following rationale: to obtain a physically stable cake after freeze-drying, a total solid content to be freeze-dried of around 3% was desired. To achieve this goal, around 1.5% of API (in combination with a co-former) was to be dissolved in pure water as well as in water-based surfactant solutions. Thereby, the initial applicability of different surfactants was investigated. API and co-former were weighed accurately and placed in a measuring tube, which contained the surfactant solution. To enhance the dissolution rate, the samples were placed in a heated sonication bath for 20 min. If dissolving a certain amount of API and the co-former led to the formation of a clear solution by visual inspection, the combination was considered soluble enough for freeze-drying. The results are shown in the Supplementary Materials (Table S1 (Supplementary Materials)).

### 2.3. Freeze-Drying

NAP was dissolved in the surfactant solution or water, respectively, with ARG or LYS at 1:1 molar ratio (1:0.76 mass ratio for ARG/1:0.63 mass ratio for LYS). To obtain clear solutions, it was necessary to adjust the pH to a value of 8 by adding 0.5 M NaOH solution. The exact amount of NaOH solution applied is included in Table S2 (Supplementary Materials) together with a complete overview of the composition of the freeze-dried samples. Three quantitatively different combinations were freeze-dried to investigate the influence of formulation parameters on the product. As an intermediate point, a 1.8% surfactant solution was prepared in which 1.5% NAP and AA (in the above-mentioned 1:1 molar ratio) were dissolved. To investigate the influence of the surfactant concentration, the latter was decreased to 0.9% whilst the absolute mass of the NAP-AA mixture was kept constant. Lastly, for the investigation of the total solid content, the ratio between NAP-AA and the surfactant was kept constant, but the surfactant concentration and the concentration of API and co-former were increased: 2.5% NAP plus ARG or LYS in 1:1 molar ratio were dissolved in a 3% surfactant solution.

The samples are referred to as NAP-AA-surfactant-concentration, where the concentrations are named high (3.0% surfactant), medium (1.8% surfactant) or low (0.9% surfactant). For the water-based samples, the low concentration was omitted.

Since different combinations of drug and excipient were screened, it was decided to use a conservative freeze-drying cycle rather than an optimized cycle for all excipients based on their *T_g_* ’ values. The samples were freeze-dried in an Alpha 1- 2 LDPlus laboratory scale freeze-dryer (Martin Christ GmbH, Osterode am Harz, Germany). The 2R freeze-drying vials (Martin Christ GmbH, Osterode am Harz, Germany) were filled with 1.0 mL of the sample solution. The samples were frozen to −35 °C over a period of three hours; subsequently, the temperature was kept constant at −35 °C for two hours. Primary drying was performed for 13 h at −20 °C. For secondary drying, the temperature was increased to 0 °C and maintained for seven hours. The pressure was kept constant at 0.370 mbar for the entire drying process and the vials were closed under vacuum at the end of the process.

### 2.4. X-Ray Powder Diffraction (XRPD)

All samples were analyzed using an X’Pert PRO X-ray diffractometer (PANalytical, Almelo, The Netherlands) with Cu Kα radiation (1.5418 Å), a current of 40 mA and an acceleration voltage of 45 kV. Samples were measured in reflectance mode in the range between 5 and 35° 2θ, with a scan rate of 0.0625° 2θ/s and a step size of 0.026° 2θ. The data collected were analyzed using X’Pert HighScore Plus (PANalytical, Almelo, The Netherlands). The reference diffractograms for all starting materials can be found in the Supplementary Materials (Figure S1 (Supplementary Materials)). The experimentally obtained diffractograms of the different NAP-ARG and NAP-LYS formulations were compared to the crystalline structures obtained by the same method from neat substances. For the NAP-ARG salt (reference code JASHOB), a diffractogram was additionally generated by using the structure from the Cambridge Crystal Data Center (Cambridge, United Kingdom) and the Mercury v3.9 software (CCDC, Cambridge, United Kingdom). A salt of NAP-LYS as a reference was prepared by solvent evaporation and analyzed with the XRPD.

### 2.5. Modulated Differential Scanning Calorimetry (mDSC) and Thermogravimetric Analysis (TGA)

Thermal analysis was performed using a Discovery DSC (TA Instruments, New Castle, DE, USA). Approximately 7–10 mg of each sample was placed in an aluminum Tzero pan and sealed with an aluminum hermetic lid. The samples were cooled down to −30 °C, kept isothermal for two minutes and then heated to 100 °C with a heating rate of 3 °C/min with an underlying modulation amplitude of 1 °C and a period of 50 s. A constant nitrogen flow rate of 50 mL/min was applied. To determine the glass transition temperature (T_g_), the data collected were analyzed using Trios software (TA instruments, New Castle, DE, USA). In addition to the mDSC, thermogravimetric analysis was carried out for each sample using the Discovery TGA (TA Instruments, New Castle, DE, USA) in order to determine the residual water content in the samples after freeze-drying. The data were analyzed using the Trios software as well.

### 2.6. Physical Stability

All samples were sealed within the freeze-dryer under vacuum. The closed vials were stored in a desiccator over silica gel at room temperature. The stability was assessed by XRPD at day 0 and after 4, 8 and 18 weeks.

## 3. Results and Discussion

### 3.1. Solubility Investigations

In order to truly follow the thermodynamic pathway in the establishment of a co-amorphous system, it is expected that both API and co-former are to be dissolved. An overview of the investigated combinations is given in the Supplementary Materials (Table S1 (Supplementary Materials)). It can be seen that numerous combinations of other APIs besides NAP and also NAP without the AA did not result in a clear solution and were thus excluded from further investigation in this initial study.

Naproxen with arginine and lysine, respectively, was chosen for further study. This choice is based on the fact that NAP-ARG has been reported for other production techniques [24] and that it was possible to obtain clear solutions for various combinations. The beneficial effect of the surfactant on the solution preparation could be practically observed by the generally lower amount of NaOH solution needed to dissolve NAP fully.

### 3.2. Solid State Analysis of the Samples

**Figure 1:**
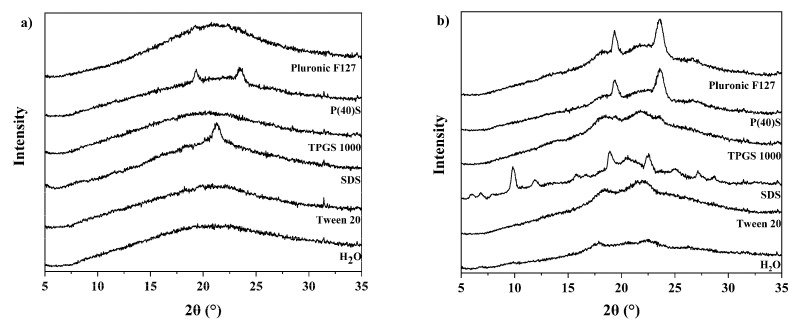
XRPD diffractograms for freshly prepared freeze-dried systems containing naproxen and arginine (NAP-ARG) (**a**) or naproxen and lysine (NAP-LYS) (**b**), respectively, both with surfactant at medium concentration.

After completion of freeze-drying, predominantly compact cakes were obtained, except for samples containing tween 20, which showed a collapsed structure. All samples were investigated by XRPD in order to determine their solid state. Generally, no reflections connected to NAP or the AA were observed, indicating the formation of an amorphous API system (Figure 1 for intermediate concentrations; see Figure S2, Supplementary Materials for low and high concentrations).

In more detail, when freeze-drying NAP-ARG samples from the medium 1.8% surfactant solution, surfactant reflections could be observed for the samples containing sodium dodecyl sulfate at 21.5° 2θ and polyoxyethylene (40) stearate at 19.5° 2θ and at 23.6° 2θ (Figure 1a). Reduction of the surfactant concentration to the low level of 0.9% removed the surfactant reflections. When the total solid content was increased to the high level, the same results as for the medium level were observed.

Comparatively more reflections were observed in freeze-dried NAP-LYS samples (Figure 1b). At the medium level, reflections were observed for sodium dodecyl sulfate, polyoxyethylene (40) stearate, pluronic F-127 and TPGS 1000. No reflections could be attributed to NAP or LYS, and all reflections were therefore again attributed to the surfactants. A reduction of the surfactant concentration to 0.9% led to the disappearance of surfactant reflections in pluoronic F-127 and to a reduction in intensity for polyoxyethylene (40) and sodium dodecyl sulfate. Increasing the total solid content to the high level showed the same results as for the medium concentration.

### 3.3. Thermal Analysis of the Amorphous Samples

Thermal analysis was used to obtain a more detailed picture of the solid state of the amorphous samples. The residual water content was determined by TGA. DSC was used to identify the T_g_ of the freeze-dried amorphous systems and to study whether a homogeneous or a heterogeneous system was obtained. The occurrence of a single T_g_ was seen as an indicator of a homogeneous system, whilst the occurrence of several T_g_s indicated a heterogeneous system.

Besides this categorization, it was also of interest to obtain some knowledge about the distribution of components in the systems. For this aim, theoretical T_g_s were calculated by applying the Fox equation [27],

(1) $$T_{g}=T_{g1}\times \omega_{1}+T_{g2}\times \omega_{2}$$

where T_g_ is the theoretical glass transition temperature of the mixture, with T_g1_ and T_g2_ being the glass transition temperatures for the isolated compounds and ω_1_ and ω_2_ the mass fractions of the two compounds in the mixture. The value for the freeze-dried co-amorphous salt was used as component 1, the freeze-dried surfactant as component 2. The obtained laboratory results were compared with the theoretical values. The T_g_s of the pure co-amorphous salts of NAP-ARG and NAP-LYS produced by ball milling were determined as a reference [5] to ensure the validity of the obtained T_g_s (Table 1). No T_g_ was detectable for T20, indicating a value below −100 °C. For comparative calculation purposes, in Table 2 and Table 3, therefore, a T_g_ of −100 °C was assumed, in the awareness that the calculated T_g_s would differ strongly from the actual value.

As a first observation, it can be seen that both homogeneous and heterogeneous systems were formed upon freeze-drying. Whilst the majority of ARG-based systems showed the occurrence of two T_g_s, only a single LYS-based system showed two T_g_s, which in addition were very close to each other (Table 2 and Table 3). This indicates that LYS based systems show a higher probability of obtaining a homogeneous ternary system, whilst ARG-based systems had a higher likelihood of resulting in a heterogeneous system. The level of surfactant had no influence on the establishment of a homogeneous versus a heterogeneous system, as identical results were obtained for all surfactant levels inside each system. The surfactant level has also only a minor influence on the value of the observed T_g_s. On the other hand, the type of surfactant in combination with the respective AA determined whether a homogeneous or a heterogeneous system was developed.

NAP-LYS systems generally resulted in homogeneous systems (Table 2). The rather low experimental T_g_ value indicates that the co-amorphous salt is not present on its own, as this would lead to a T_g_ in the area of 65°C. The obtained values are also in agreement with the theoretical values of the homogeneous systems and it can therefore be assumed that a ternary homogeneous system has formed of the NAP-LYS salt with the surfactants. Only for the samples of NAP-LYS with TPGS were two T_g_s observed in close proximity to each other. Both values are too low to correspond to the pure salt; therefore, both phases must contain all three ingredients (and the residual moisture) but in different concentrations, meaning that there is one surfactant-rich and one surfactant-poor fraction. The exact location of the residual moisture in between these phases cannot be determined due to the small difference between the T_g_ values.

**Figure 2:**
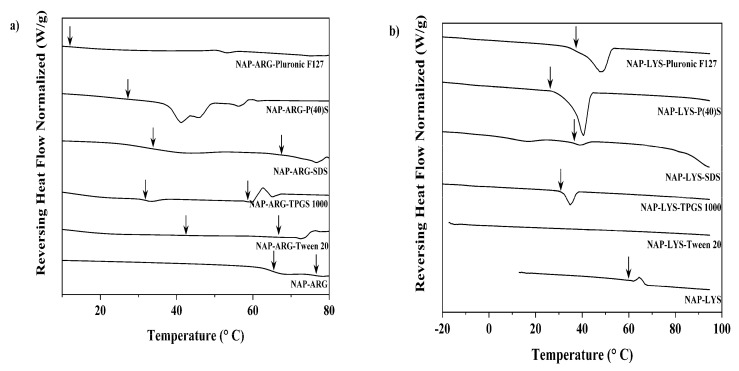
DSC results with T_g_s marked with an arrow for all compositions of naproxen and arginine (NAP-ARG) (**a**) or naproxen and lysine (NAP-LYS) (**b**), both with surfactant in the medium concentration.

The situation in ARG-based systems was in general more complex than in LYS-based systems, due to the frequent occurrence of a second T_g_. In ARG-based systems with T20, TPGS and SDS with two T_g_s, the highest T_g_ was located close to the T_g_ of the NAP-ARG salt (Figure 2a) and was therefore attributed to a pure NAP-ARG amorphous salt phase. The lower T_g_ was less unambiguous but it can be assumed that this T_g_ is based on a phase consisting of NAP-ARG salt, the surfactants and the majority of the residual water.

The observed second T_g_ can be explained by several theoretical combinations. It should be considered that the presence of water as a component gives four different components that may or may not interact with each other, both completely or partially. Therefore, thermal analysis can only give some indications of the molecular arrangement of these rather complex systems. When interpreting the thermal data, it has to be considered that NAP-AA systems usually have a much higher T_g_ than the individual components, due the formation of an amorphous salt. Either the surfactant, water or both can then interact with this salt or might even prevent the formation of the salt. Therefore, a qualitative approach to the interpretation of the T_g_s values is the first step; this step includes the proximity to the T_g_ of the salt as well as the distance between the two observed T_g_s. In order to obtain some insight into the composition of the phases, a comparison between the T_g_s of NAP-AA co-amorphous salts and the T_g_s of NAP-AA-surfactant-co-amorphous systems might be helpful. All the used surfactants have T_g_s below zero degrees Celsius, far below the NAP-AA-salts. According to the Fox equation, the samples including surfactants would be calculated to show T_g_s far lower than the pure salts, with the exact decrease dependent on the corresponding surfactant.

Table 3 gives an overview over the obtained results. Two of the surfactants, namely P40(s) and PF 127, yield homogeneous systems with a rather low T_g_, as in the majority of the NAP-LYS systems described above. It can therefore be assumed that these two surfactants also led to the formation of a homogeneous ternary system. The other three surfactants on the other hand lead to the formation of a heterogeneous system with two T_g_s. In contrast to LYS, where these two T_g_s were rather close together, ARG-based systems show a large difference between the T_g_s. The higher Tg is in the area of the NAP-ARG co-amorphous salt and therefore attributed to this component, as also discussed in connection with Figure 2. The lower T_g_ value is mostly found in the area where a homogenous system would be expected to have a T_g_. It can therefore be concluded that, upon freeze-drying NAP-ARG together with the other three surfactants, phase separation occurs, leading to the formation of a co-amorphous NAP-ARG salt and a ternary mixture of the salt and the surfactant.

As a conclusion to the thermal investigation, it can be stated that freeze-drying of the investigated systems resulted in the formation of co-amorphous NAP-AA salts. Both homogeneous and heterogeneous systems were obtained, and an interplay between surfactant type and co-former AA determined whether a homogeneous or a heterogeneous system was obtained. PF127 and P40(s) resulted in homogenous systems in both cases, whilst TPGS resulted in a heterogeneous system in both cases, albeit with different phase composition characteristics. The remaining two surfactants, SDS and T20, resulted in homogeneous systems with NAP-LYS but heterogeneous systems with NAP-ARG.

### 3.4. Stability Assessment

Multiple samples of each NAP-AA-surfactant composition were stored over several weeks to be compared with the freshly produced freeze-dried products by XRPD analysis. It was observed that those surfactant reflections, which were already seen in the fresh samples, partly increased in their intensity. Furthermore, reflections originating from crystalline NAP-ARG salt, NAP-LYS salt, crystalline NAP and ARG, respectively, became visible in a few samples. It was earlier shown that the various surfactants were differently successful in obtaining both an amorphous as well as a homogeneous system. Due to the observed differences it was expected that also storage stabilization efficacy would be different. It was thus studied to which degree the different surfactants had a beneficial effect on the physical stability of the co-amorphous systems and in addition the influence of the total solid amount and the ratio of the three components, respectively. It was clearly visible that the two amino acid co-formers resulted in different levels of storage stability. Both NAP-AA systems without surfactant recrystallized before 4–8 weeks of storage, thereby showing reduced storage stability compared with an earlier study of the same system, which reported 10 weeks of stability, although for a system produced by dry ball milling [26]. This reduced stability might be attributed to the remaining water content in the sample or other sources to induce nucleation, such as dust particles incorporated before the freeze-drying operation.

Whilst NAP-LYS without surfactant showed recrystallisation after 8 weeks, the majority of surfactant-containing systems showed increased physical stability. The composition of NAP-LYS-SDS developed slight peaks representing the formation of recrystallized NAP-LYS salt. The systems with the other four surfactants, i.e., T20, TPGS, P40S and PF127, remained amorphous over the whole storage duration of 18 weeks. Comparing these findings with the T_g_ values in Table 2, it can be seen that the surfactant systems show higher stability despite a lower T_g_, which might be surprising at first. However, this behavior can be explained by the surfactants preventing recrystallization by local separation of the drug and AA molecules, corresponding to the stabilization mechanism of polymeric amorphous solid dispersions.

However, a different picture was obtained for NAP-ARG systems. NAP-ARG combined with tween 20, at medium and high concentrations of the ingredients, recrystallized in a similar time frame to the samples without surfactant, whilst a low concentration of tween stabilized the system. This can be speculated to be due to two counteracting effects, namely destabilization by the low theoretical T_g_ of the surfactant (dominating at medium and high concentrations) and inhibition of recrystallization due to spatial separation (dominating at the low concentration). This hypothesis can be strengthened by the fact that this behavior is especially observed with tween, which possesses the lowest T_g_ of the investigated surfactants. Only NAP-ARG systems with SDS (and P40(S) medium) remained amorphous for the whole duration of the stability study. All other samples recrystallized at varying time points during the study. The recrystallization tendency does not seem to be connected with the occurrence of a homogeneous or heterogeneous system, but some influence of the surfactant type can be observed.

**Figure 3:**
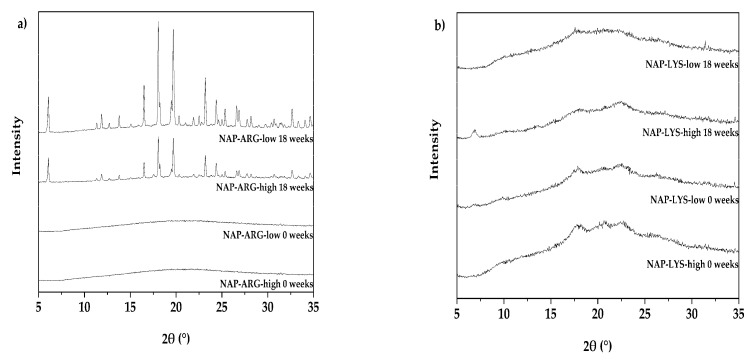
Physical stability by XRPD for different naproxen-amino acid (NAP-AA) salts comparing fresh samples (0 weeks) and stored samples (18 weeks), naproxen-arginine (NAP-ARG) salts (**a**) and naproxen-lysine (NAP-LYS) salts (**b**).

After 18 weeks of storage, the tendencies already seen after four weeks were confirmed. All samples containing only NAP-AA were classified as crystalline. While NAP-ARG (Figure 3a) samples showed clear reflections of the pure NAP and ARG indicating high crystallinity, the NAP-LYS samples (Figure 3b) were still mostly amorphous but with indications for the recrystallization of the salt. The complete data set for the solid-state stability after 18 weeks is included in the Supplementary Materials (Figure S3).

Out of the samples that were combined with a surfactant, almost all the NAP-ARG samples showed recrystallization under storage, except for the combinations of NAP-ARG-SDS in all concentrations and the medium concentration of NAP-ARG-P40S.

**Figure 4:**
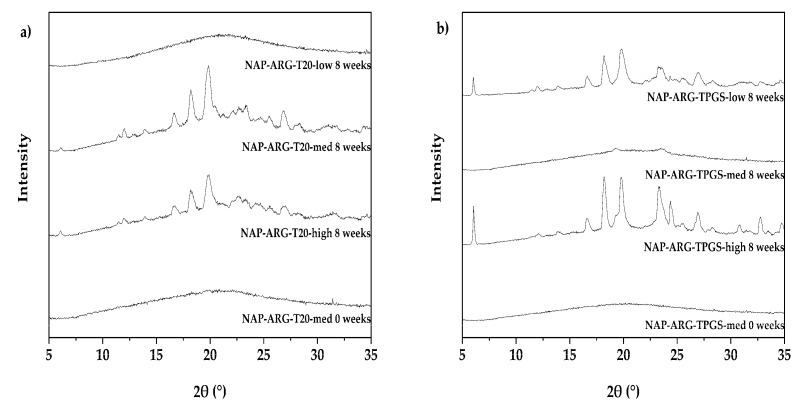
Physical stability by XRPD for different naproxen-arginine (NAP-ARG) salts: NAP-ARG-Tween 20 (**a**) showing improved stability with a low concentration of surfactant after 8 weeks, NAP-ARG-TPGS (**b**) showing an optimum for surfactant at the medium concentration.

Taking a closer look into the influence of the total solid compound and the concentration of the surfactant in the samples, no clear statement can be made as all surfactants behave differently. SDS and PF 127 showed the same stability independent of the formulation parameters. On the other hand, the lowest solid content formulations showed the highest stability for SDS NAP-ARG-T20 (Figure 4a). No salt-associated reflections appeared at the lower concentration, but such reflections developed after four weeks at the medium and high concentrations. On the other hand, an indication for an optimal concentration of surfactant in order to gain a beneficial effect on storage stability can be seen for the NAP-ARG-TPGS systems after 8 weeks (Figure 4b), where only the medium concentration remained amorphous. A similar tendency can be observed for P40(S). A detailed mechanistic understanding of the reasons that the various surfactants behave differently is outside of the scope of this initial study, but for future applications, it should be considered that the surfactant concentration might need to be adjusted depending on the system based on the assumption of the two counteracting effects described earlier (spatial separation versus reduced T_g_). Generally, a high total solid content seems to negatively influence the storage stability: this can easily be explained by the fact that higher concentrations provide a higher likelihood for the formation of nuclei, which then can facilitate crystal growth. On the other hand, the physical instability of a low concentrated system might be due to collapse-induced changes in the system, enabling recrystallization.

In summary, this initial study showed the feasibility of freeze-drying for the production of co-amorphous drug–AA systems applying surfactants as additional excipients. Whilst an initial insight into the formation and stability of the systems was obtained, showing the influence of both surfactant type and surfactant amount, as well as the differences between two co-amorphous systems, several points still need further attention, such as a mechanistic understanding of the mode of action of the surfactants, the influence of the freezing protocol for product optimization and finally the dissolution behavior of the obtained systems.

## 4. Conclusions

This study showed that a co-amorphous naproxen-arginine and naproxen-lysine system can be obtained by freeze-drying. The inclusion of different surfactants into the co-amorphous system has been accomplished successfully and several surfactants were found to increase the likelihood of maintaining a co-amorphous system over a period of at least eighteen weeks. Lysine-based systems were found to be more stable than arginine-based systems. However, the presence of surfactants showed varying influence, as the formation of both homogeneous and heterogeneous systems was mainly determined by the type of surfactant present. An increase in the total solid content increases the likelihood of crystallization for NAP-ARG samples. An increase in the concentration of the surfactant led to the appearance of surfactant reflections; however, the rising total content of the surfactant cannot be directly linked to the recrystallization rate in the systems. Finally, it can be stated that freeze-drying might prove to be a viable production technique for co-amorphous systems, although it might only be applicable for a selection of compounds. In addition, the role of surfactants in co-amorphous systems in general needs further elucidation.

## Figures and Tables

**Table 1:** T_g_s of the amorphous ball milled salts and the surfactants.

System	NAP-ARG	NAP-LYS	SDS	T20	TPGS	P (40) S	PF127
**T_g_ (°C)**	65	59	−9.1	<−100	−24.1	−6.6	−66.5

**Table 2:** Experimentally determined T_g_s for naproxen-lysine (NAP-LYS) co-amorphous systems and water content of the systems. Re-calculated T_g_ for the systems without water, theoretical T_g_ for a homogenous system and storage stability as duration until first recrystallization. Calculated values for Tween 20 systems marked with * must be regarded as estimates. n.d. = not detectable.

	Experimental			Calculated			
Surfactant System	T_g_ 1 (°C)	T_g_ 2 (°C)	Water Content (w %)	T_g_ 1 (°C)	T_g_ 2 (°C)	Theoretical T_g_ of Homogenous System	Duration until Recrystallization (weeks)
**H_2_O-med**	60		3.5	67			8
**H_2_O-high**	64		3.1	70			8
**SDS-low**	38		2.0	42		41	4
**SDS-med**	39		1.0	40		30	4
**SDS-high**	36		0.9	38		30	4
**P40S-low**	27		2.1	31		41	>18
**P40S-med**	25		1.4	27		31	>18
**P40S-high**	28		0.3	28		31	>18
**PF127-low**	41		0.5	42		25	>18
**PF127-med**	36		0.5	37		6	>18
**PF127-high**	37		0.8	38		6	>18
**T20-low**	n.d.		1.3	n.d.		16 *	>18
**T20-med**	n.d.		1.2	n.d.		−8 *	>18
**T20-high**	n.d.		0.8	n.d.		−8 *	>18
**TPGS-low**	27	34	1.5	30	37	37	>18
**TPGS-med**	29	34	0.8	30	36	24	>18
**TPGS-high**	29	33	0.9	31	35	24	>18

**Table 3:** Experimentally determined T_g_s for naproxen-arginine (NAP-ARG) co-amorphous systems and water content of the systems. Re-calculated T_g_ for the systems without water, theoretical T_g_ for a homogenous system and storage stability as duration until first recrystallization. Calculated values for Tween 20 systems marked with * must be regarded as estimates.

	Experimental			Calculated			
Surfactant System	T_g_ 1 (°C)	T_g_ 2 (°C)	Water Content (w %)	T_g_ 1 (°C)	T_g_ 2 (°C)	Theoretical T_g_ of Homogenous System	Duration until Recrystallization (weeks)
**H_2_O-med**	65	76	5.0	76	87		4
**H_2_O-high**	60	81	2.2	64	86		8
**T20-low**	33	67	2.5	37	72	23 *	18
**T20-med**	43	68	1.2	45	71	−2 *	4
**T20-high**	46	69	1.3	48	72	−2 *	4
**TPGS-low**	32	58	2.3	36	63	42	8
**TPGS-med**	33	58	1.7	36	61	29	18
**TPGS-high**	34	57	1.4	37	60	29	8
**SDS-low**	38	65	2.0	42	70	46	>18
**SDS-med**	33	66	1.5	36	69	35	>18
**SDS-high**	42	65	1.6	44	68	35	>18
**P40S-low**	14		2.2	18		47	18
**P40S-med**	27		2.1	31		36	>18
**P40S-high**	38		2.0	41		36	18
**PF127-low**	23		1.1	24		32	8
**PF127-med**	16		0.7	17		12	8
**PF127-high**	24		1.9	27		12	8

## Supplementary Materials

The following are available online at https://www.mdpi.com/1999-4923/12/10/941/s1, Table S1: Solubility of different APIs in combination with surfactant and AA, Table S2: Overview of all compositions freeze-dried for further investigations, Figure S1: Solid state of starting materials, Figure S2: Solid state of freeze-dried concentrations at low and high concentration of detergents, Figure S3: Solid state upon 18 weeks of storage.
