# Supplementary material for: Preparation of Co-Amorphous Systems by Freeze-Drying
Source: Pharmaceutics. 2020 Sep 30;12(10):941. doi: 10.3390/pharmaceutics12100941 (PMC7599809; doi:10.3390/pharmaceutics12100941)
Supplement: Supplementary file 1 [file pharmaceutics-12-00941-s001.pdf]

pharmaceutics

Supplementary Materials: Preparation of Co-Amorphous Systems by Freeze-Drying

Melvin Wostry, Hanna Plappert and Holger Grohganz *

Table S1. of different APIs in combination with DET and AA, only NAP showed an improved dissolution behavior in the combination with the AA ARG, therefore NAP was used for further investigations.

**Solvent**		**Carbamazepin**	**Carvedilol**	**Naproxen**
**SDS**	**+ PEG**	No	No	No
**Tween 20**	**+ PEG**	No	No	No
**TPGS**	**+ PEG**	No	No	No
**Pluronic F127**	**+ PEG**	No	No	No
**+ Amino acid in H_2O**		-	-	Yes (with ARG)
**SDS + AA**		No	No	-
**Tween 20 + AA**		No	No	Yes (with ARG)
**TPGS + AA**		No	No	Yes (with ARG)
**Pluronic F127**		No	No	-
**Ansätze mit Urea**		No	No	No

Table S2. Overview of all compositions freeze-dried for further investigations.

**Name**	**API**	**Conc. (w/v)**	**AA**	**Conc. (w/v)**	**Surfactant**	**Conc. (w/v)**	**pH correction 0.5 M NaOH [µL]**
LYS-H_2O-med	NAP	1.5 %	LYS	0.95 %	-	-	150
LYS-H_2O-high	NAP	2.5 %	LYS	1.58 %	-	-	100
LYS-SDS-low	NAP	1.5 %	LYS	0.95 %	SDS	0.9 %	100
LYS-SDS-med	NAP	1.5 %	LYS	0.95 %	SDS	1.8 %	100
LYS-SDS-high	NAP	2.5 %	LYS	1.58 %	SDS	3.0 %	100
LYS-P40S-low	NAP	1.5 %	LYS	0.95 %	P40S	0.9 %	200
LYS-P40S-med	NAP	1.5 %	LYS	0.95 %	P40S	1.8 %	100
LYS-P40S-high	NAP	2.5 %	LYS	1.58 %	P40S	3.0 %	100
LYS-PF127-low	NAP	1.5 %	LYS	0.95 %	PF127	0.9 %	150
LYS-PF127-med	NAP	1.5 %	LYS	0.95 %	PF127	1.8 %	100
LYS-PF127-high	NAP	2.5 %	LYS	1.58 %	PF127	3.0 %	150
LYS-T20-low	NAP	1.5 %	LYS	0.95 %	T20	0.9 %	100
LYS-T20-med	NAP	1.5 %	LYS	0.95 %	T20	1.8 %	100
LYS-T20-high	NAP	2.5 %	LYS	1.58 %	T20	3.0 %	100
LYS-TPGS-low	NAP	1.5 %	LYS	0.95 %	TPGS	0.9 %	100
LYS-TPGS-med	NAP	1.5 %	LYS	0.95 %	TPGS	1.8 %	100
LYS-TPGS-high	NAP	2.5 %	LYS	1.58 %	TPGS	3.0 %	150
ARG-H_2O-med	NAP	1.5 %	ARG	1.13 %	-	-	150
ARG-H_2O-high	NAP	2.5 %	ARG	1.89 %	-	-	600
ARG-T20-low	NAP	1.5 %	ARG	1.13 %	T20	0.9 %	100
ARG-T20-med	NAP	1.5 %	ARG	1.13 %	T20	1.8 %	100
ARG-T20-high	NAP	2.5 %	ARG	1.89 %	T20	3.0 %	200

ARG-TPGS-low	NAP	1.5 %	ARG	1.13 %	TPGS	0.9 %	100
ARG-TPGS-med	NAP	1.5 %	ARG	1.13 %	TPGS	1.8 %	100
ARG-TPGS-high	NAP	2.5 %	ARG	1.89 %	TPGS	3.0 %	100
ARG-SDS-low	NAP	1.5 %	ARG	1.13 %	SDS	0.9 %	100
ARG-SDS-med	NAP	1.5 %	ARG	1.13 %	SDS	1.8 %	200
ARG-SDS-high	NAP	2.5 %	ARG	1.89 %	SDS	3.0 %	100
ARG-P40S-low	NAP	1.5 %	ARG	1.13 %	P40S	0.9 %	100
ARG-P40S-med	NAP	1.5 %	ARG	1.13 %	P40S	1.8 %	100
ARG-P40S-high	NAP	2.5 %	ARG	1.89 %	P40S	3.0 %	200
ARG-PF127-low	NAP	1.5 %	ARG	1.13 %	PF127	0.9 %	150
ARG-PF127-med	NAP	1.5 %	ARG	1.13 %	PF127	1.8 %	100
ARG-PF127-high	NAP	2.5 %	ARG	1.89 %	PF127	3.0 %	300

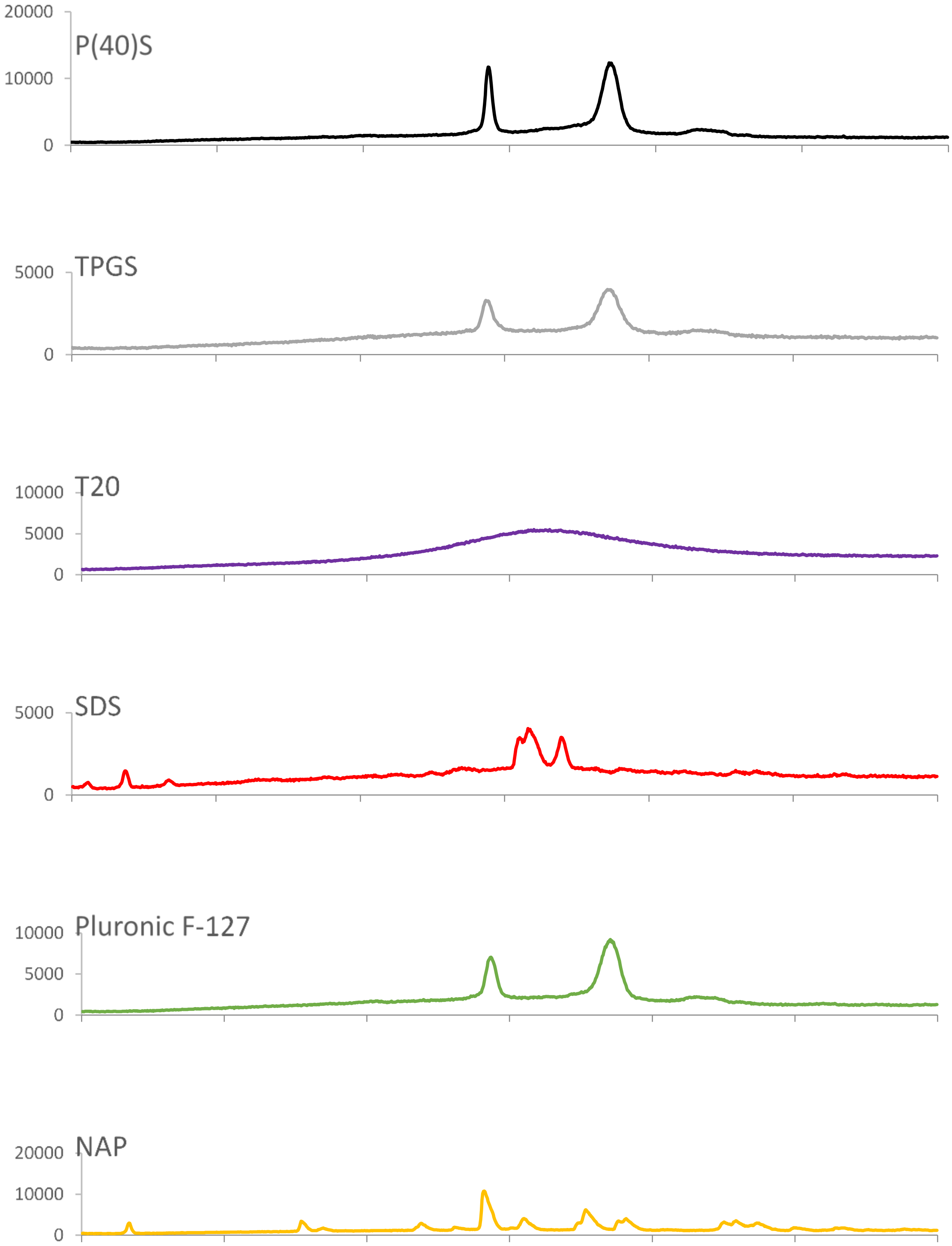

20000
10000
0
P(40)S
5000
0
TPGS
10000
5000
0
T20
5000
0
SDS
10000
5000
0
Pluronic F-127
20000
10000
0
NAP

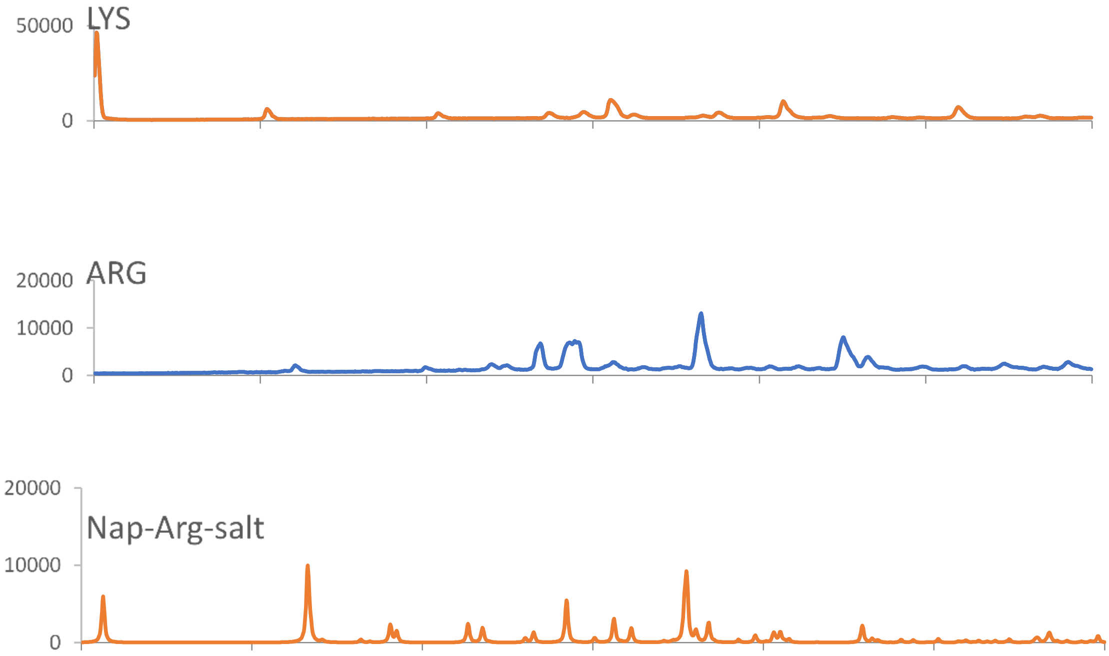

Figure S1. Solid state of starting materials Figure S2: Solid state of freeze-dried concentrations at low and high concentration of detergents.

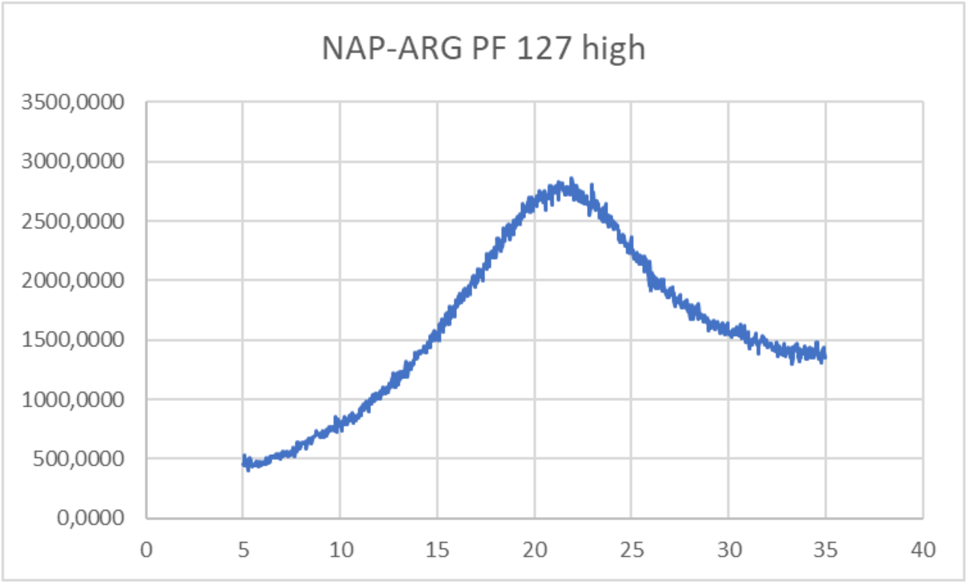

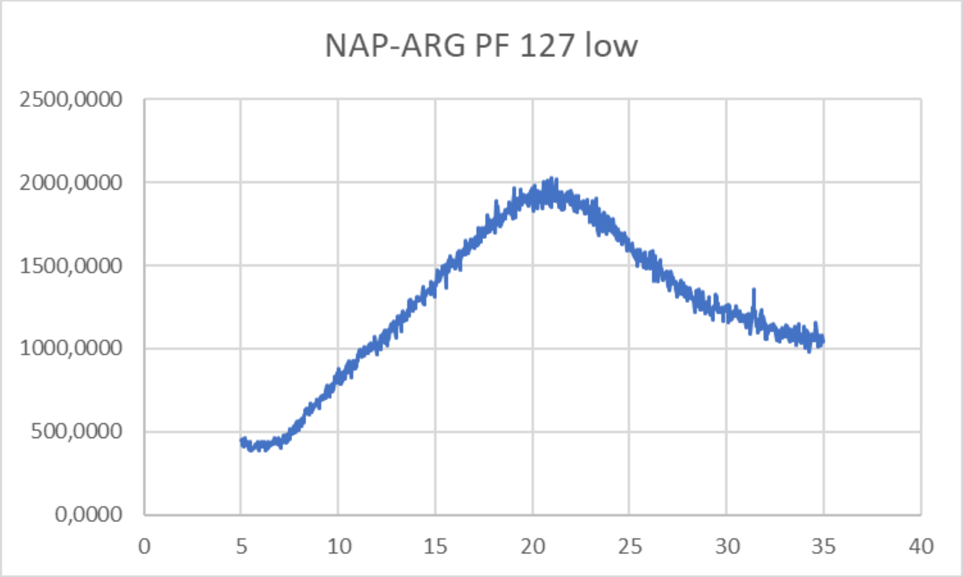
NAP-ARG PF 127 low
2500,0000
2000,0000
1500,0000
1000,0000
500,0000
0,0000
0
5
10
15
20
25
30
35
40

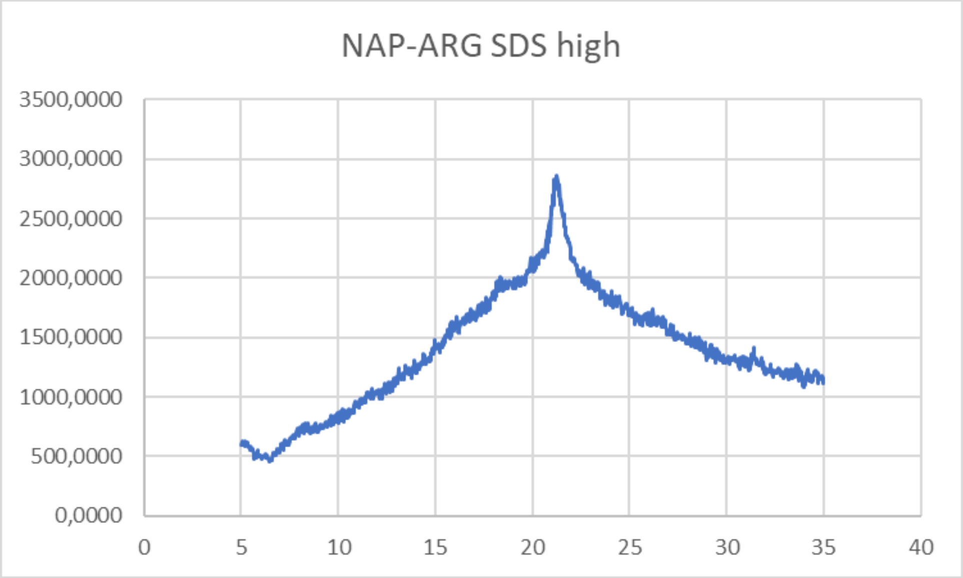
NAP-ARG SDS high
3500,0000
3000,0000
2500,0000
2000,0000
1500,0000
1000,0000
500,0000
0,0000
0
5
10
15
20
25
30
35
40

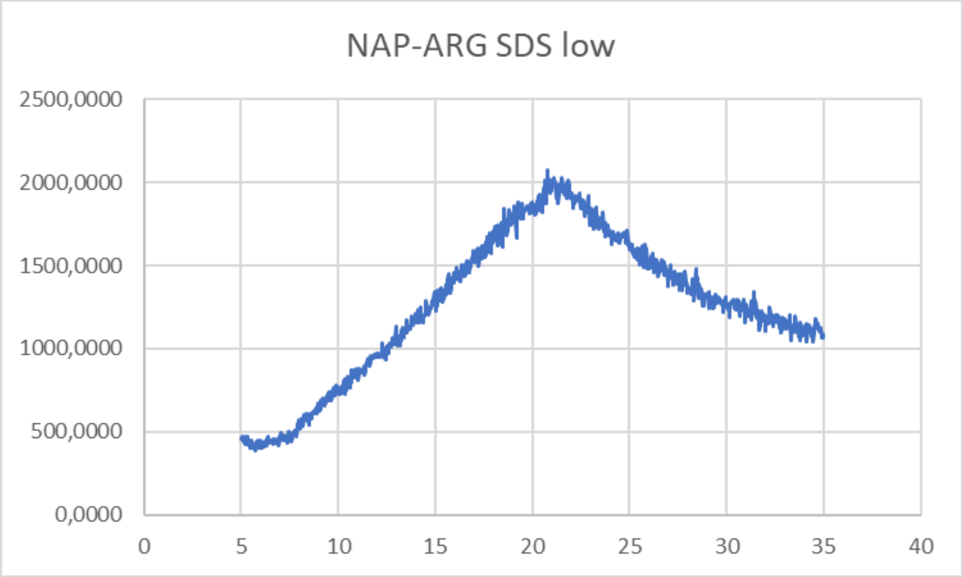
NAP-ARG SDS low
2500,0000
2000,0000
1500,0000
1000,0000
500,0000
0,0000
0
5
10
15
20
25
30
35
40

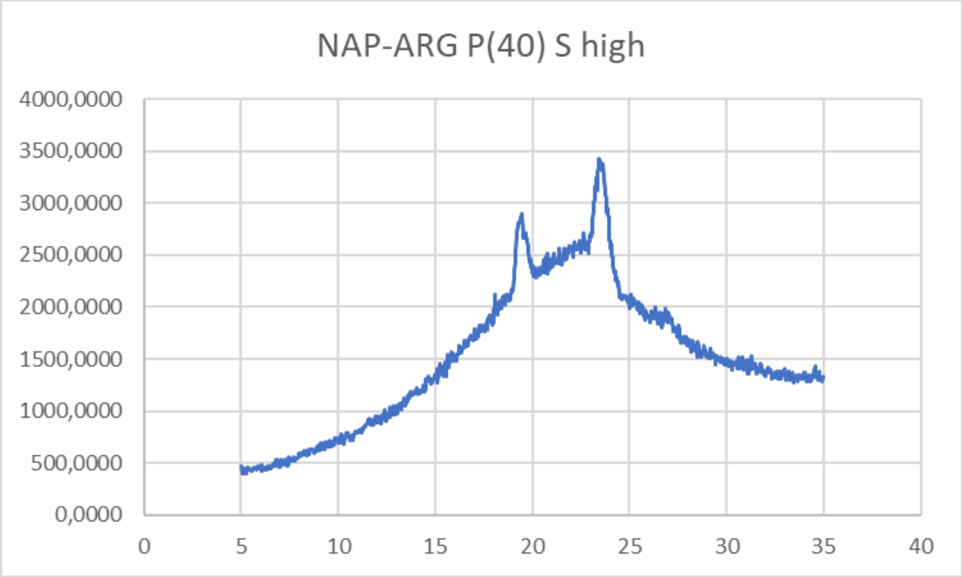
NAP-ARG P(40) S high
4000,0000
3500,0000
3000,0000
2500,0000
2000,0000
1500,0000
1000,0000
500,0000
0,0000
0
5
10
15
20
25
30
35
40

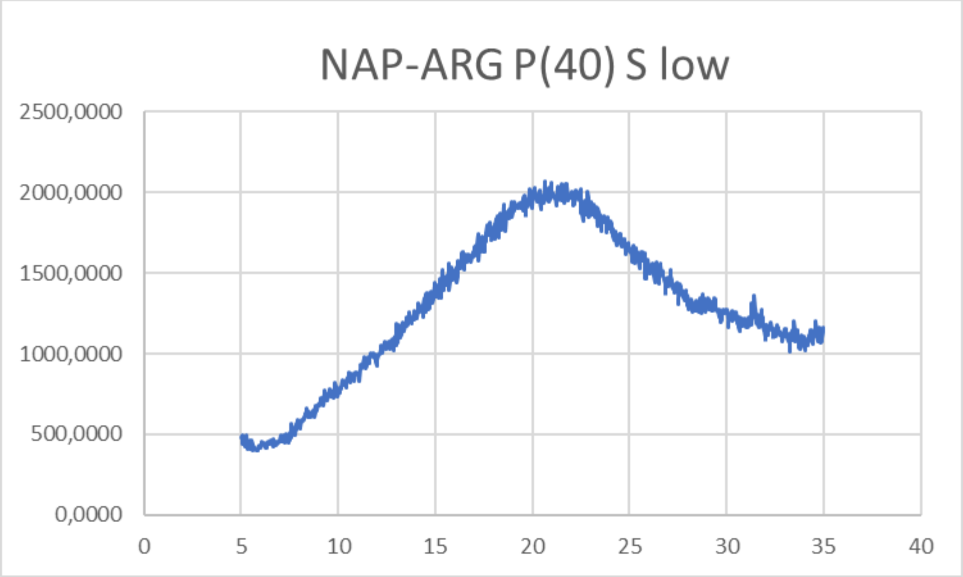
NAP-ARG P(40) S low
2500,0000
2000,0000
1500,0000
1000,0000
500,0000
0,0000
0
5
10
15
20
25
30
35
40

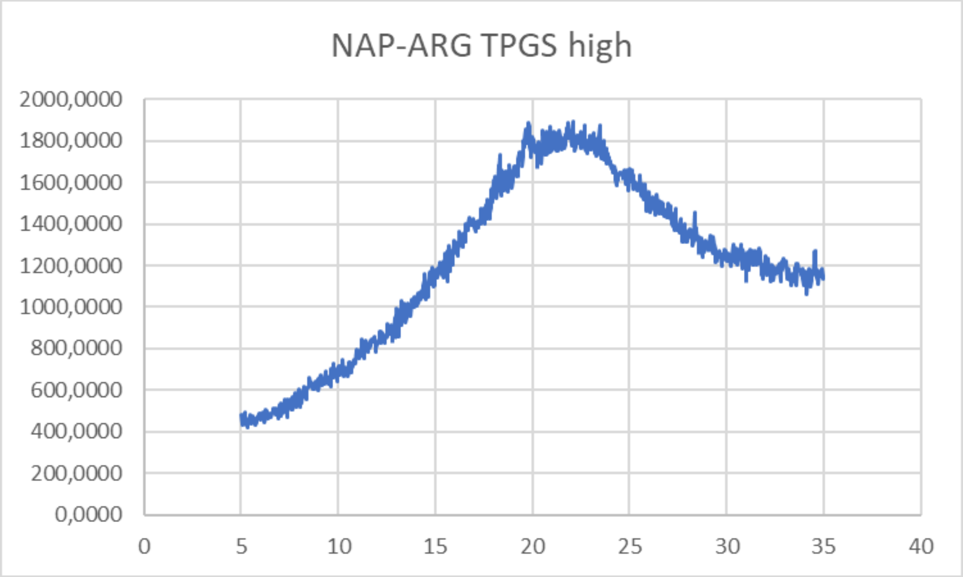
NAP-ARG TPGS high
2000,0000
1800,0000
1600,0000
1400,0000
1200,0000
1000,0000
800,0000
600,0000
400,0000
200,0000
0,0000
0
5
10
15
20
25
30
35
40

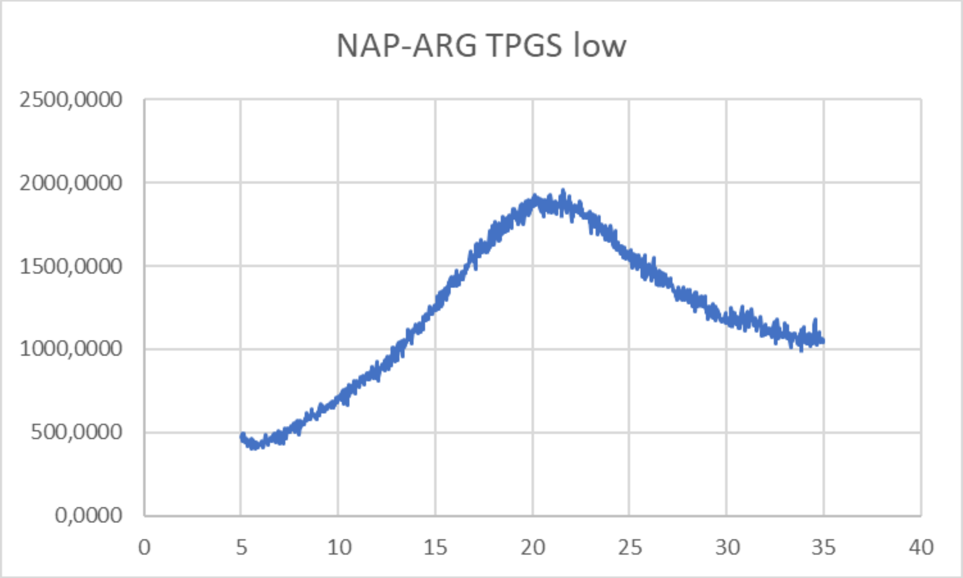
NAP-ARG TPGS low
2500,0000
2000,0000
1500,0000
1000,0000
500,0000
0,0000
0
5
10
15
20
25
30
35
40

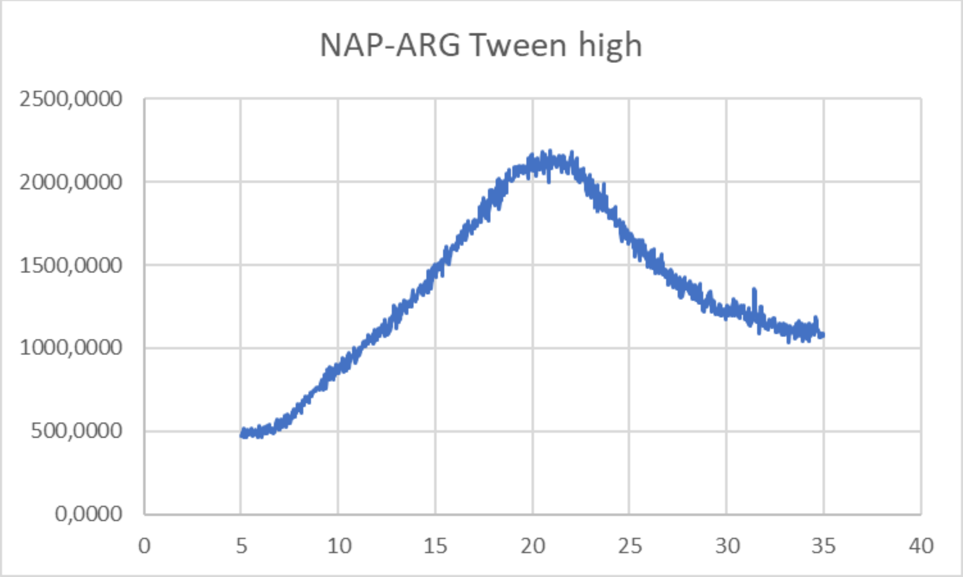
NAP-ARG Tween high
2500,0000
2000,0000
1500,0000
1000,0000
500,0000
0,0000
0
5
10
15
20
25
30
35
40

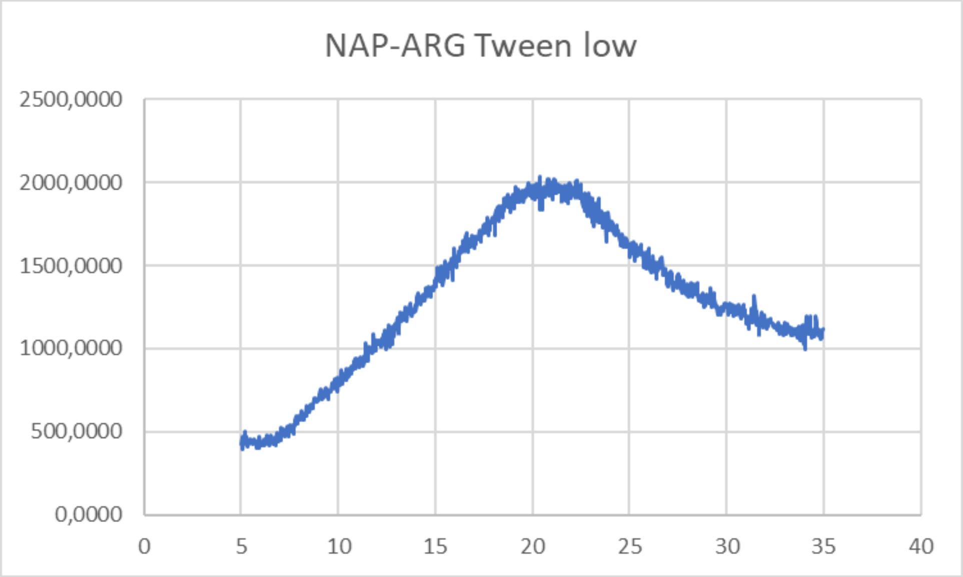
NAP-ARG Tween low
2500,0000
2000,0000
1500,0000
1000,0000
500,0000
0,0000
0
5
10
15
20
25
30
35
40

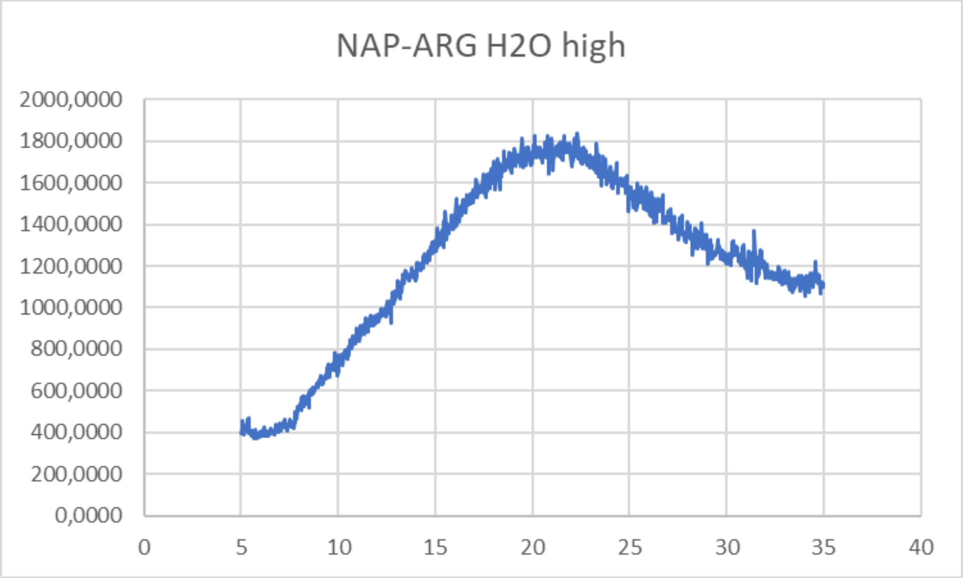
NAP-ARG H2O high
2000,0000
1800,0000
1600,0000
1400,0000
1200,0000
1000,0000
800,0000
600,0000
400,0000
200,0000
0,0000
0
5
10
15
20
25
30
35
40

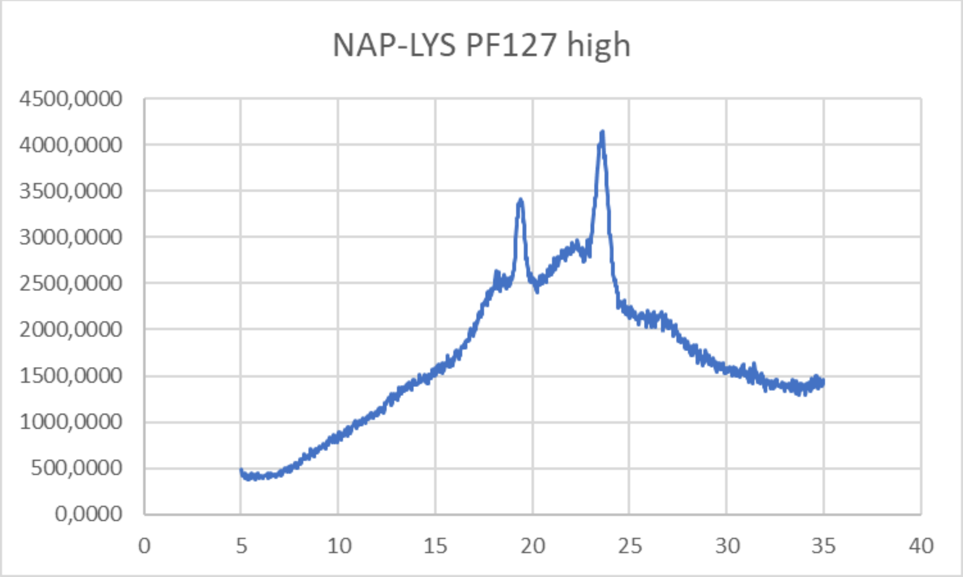
NAP-LYS PF127 high
4500,0000
4000,0000
3500,0000
3000,0000
2500,0000
2000,0000
1500,0000
1000,0000
500,0000
0,0000
0
5
10
15
20
25
30
35
40

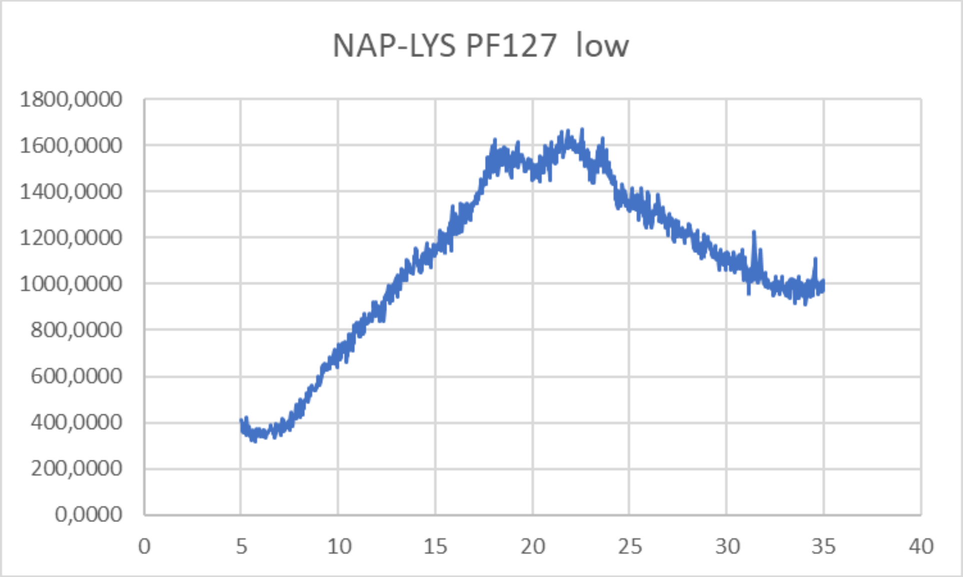
NAP-LYS PF127 low
1800,0000
1600,0000
1400,0000
1200,0000
1000,0000
800,0000
600,0000
400,0000
200,0000
0,0000
0 5 10 15 20 25 30 35 40

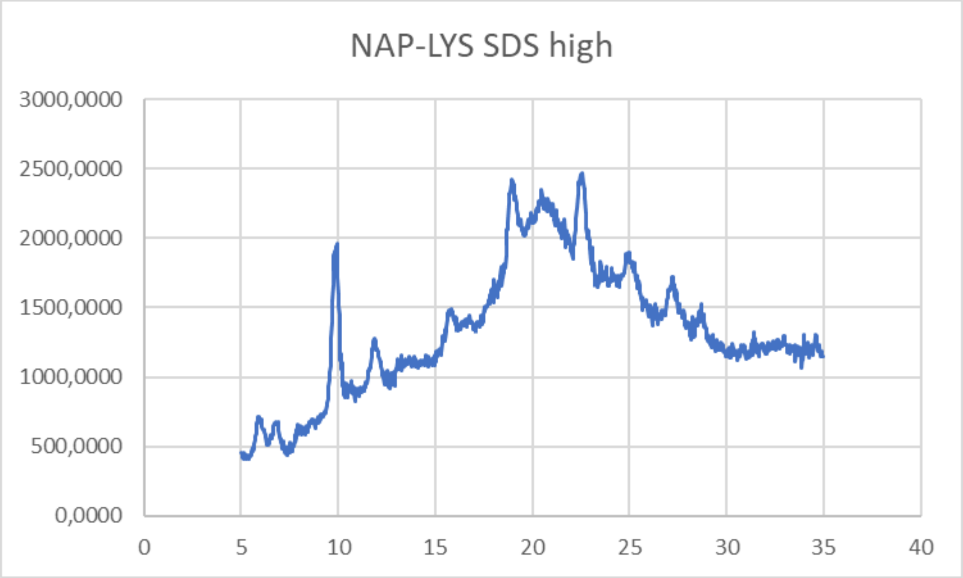
NAP-LYS SDS high
3000,0000
2500,0000
2000,0000
1500,0000
1000,0000
500,0000
0,0000
0 5 10 15 20 25 30 35 40

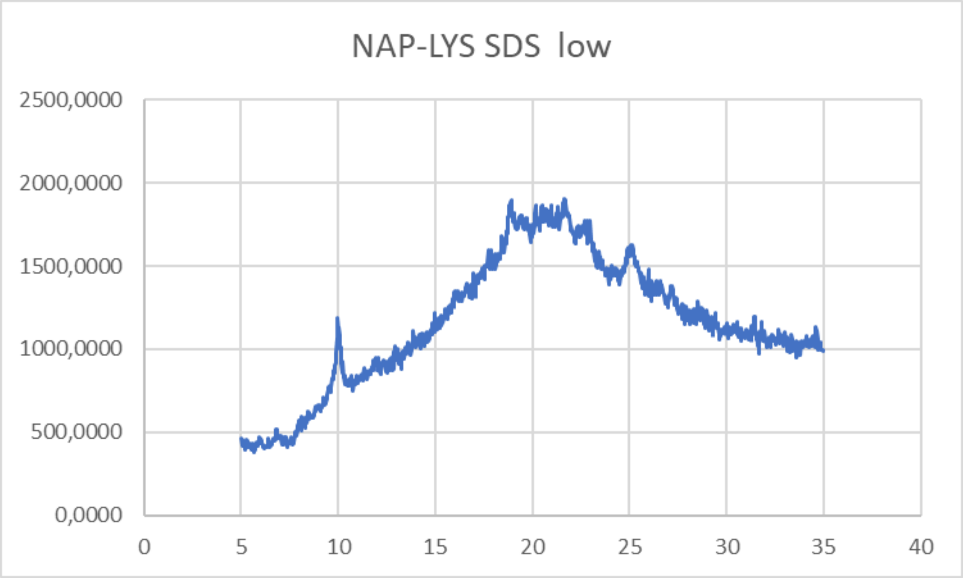
NAP-LYS SDS low
2500,0000
2000,0000
1500,0000
1000,0000
500,0000
0,0000
0 5 10 15 20 25 30 35 40

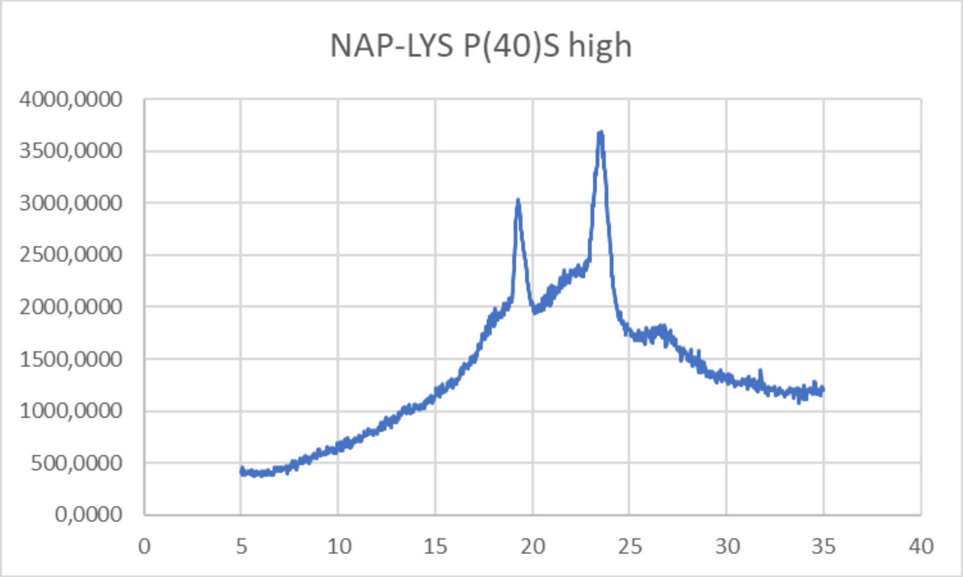
NAP-LYS P(40)S high
4000,0000
3500,0000
3000,0000
2500,0000
2000,0000
1500,0000
1000,0000
500,0000
0,0000
0
5
10
15
20
25
30
35
40

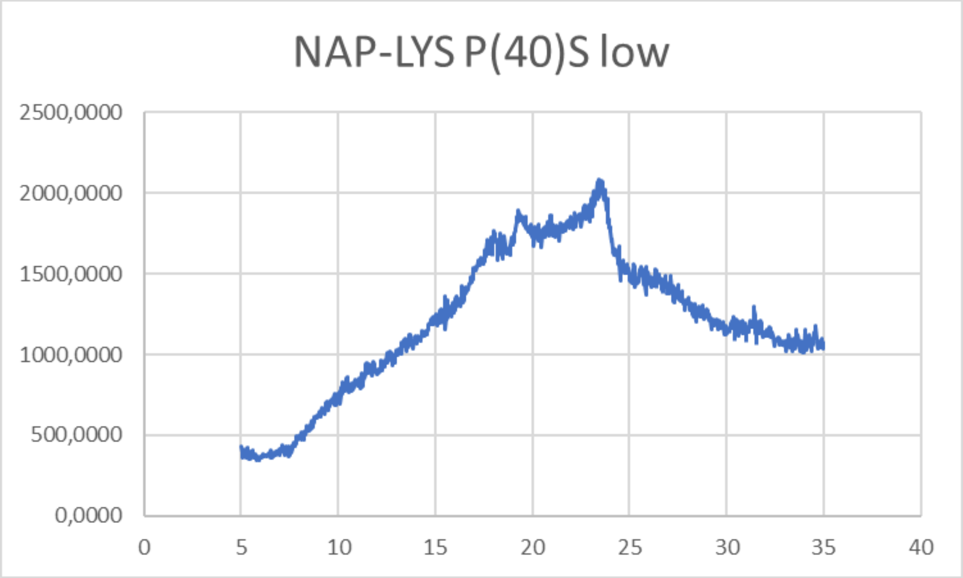
NAP-LYS P(40)S low
2500,0000
2000,0000
1500,0000
1000,0000
500,0000
0,0000
0
5
10
15
20
25
30
35
40

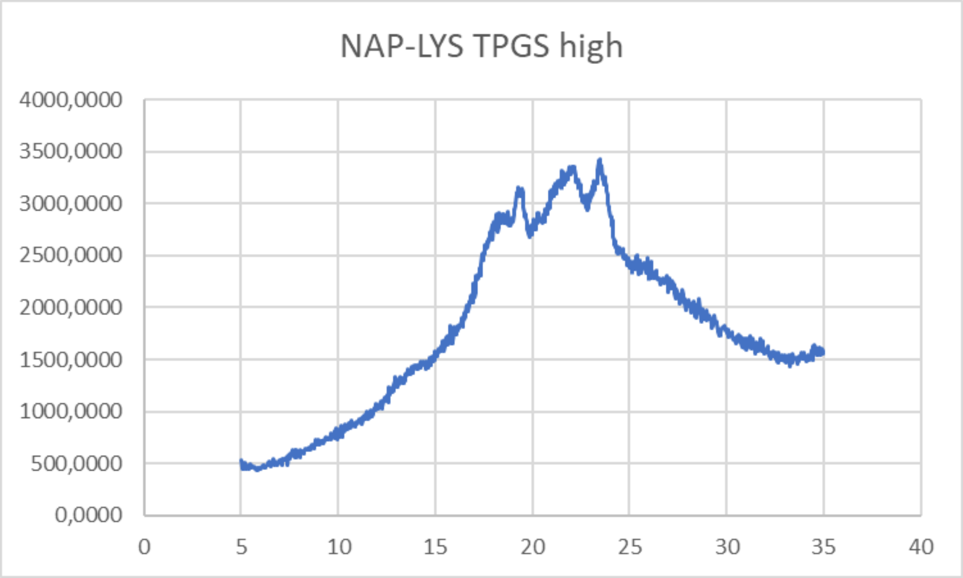
NAP-LYS TPGS high
4000,0000
3500,0000
3000,0000
2500,0000
2000,0000
1500,0000
1000,0000
500,0000
0,0000
0
5
10
15
20
25
30
35
40

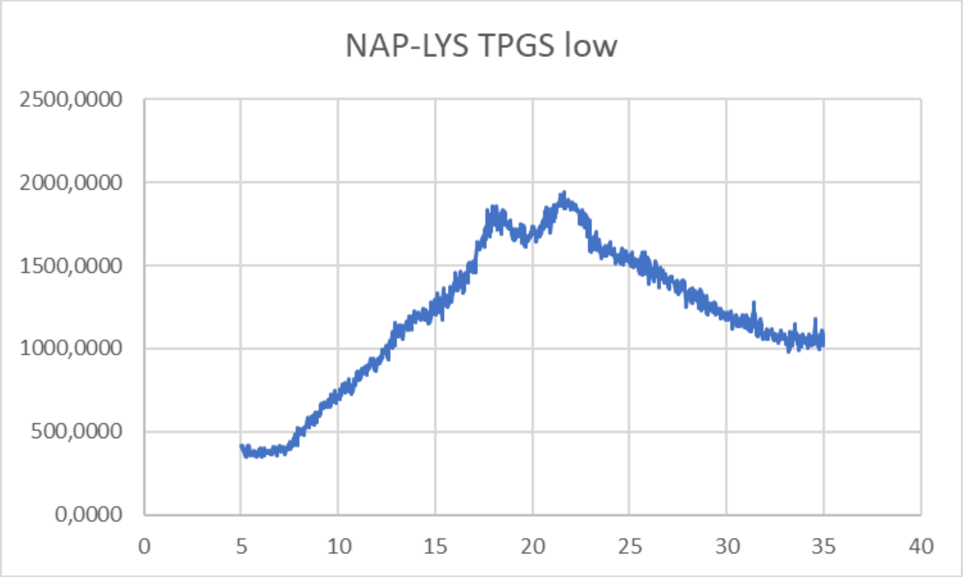
NAP-LYS TPGS low
2500,0000
2000,0000
1500,0000
1000,0000
500,0000
0,0000
0
5
10
15
20
25
30
35
40

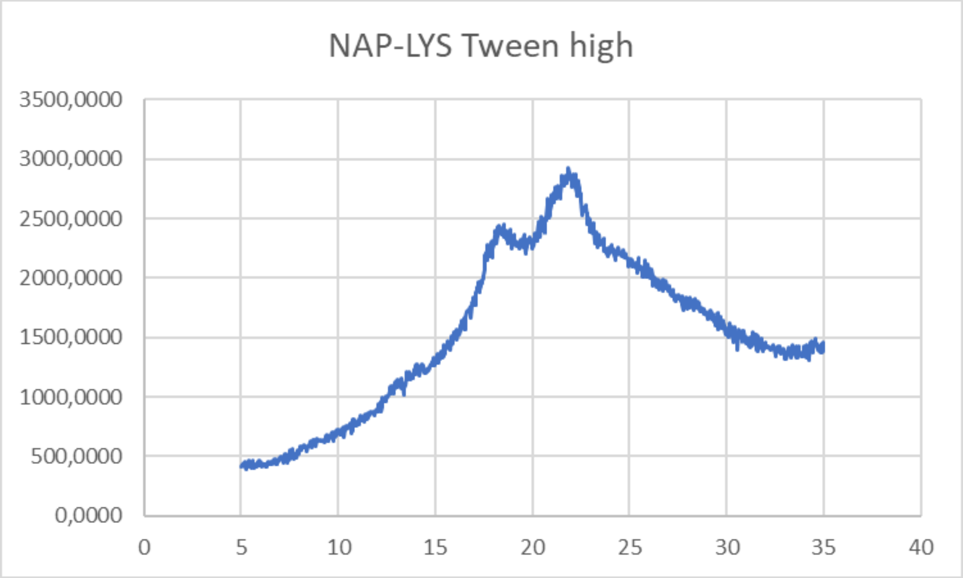
NAP-LYS Tween high
3500,0000
3000,0000
2500,0000
2000,0000
1500,0000
1000,0000
500,0000
0,0000
0
5
10
15
20
25
30
35
40

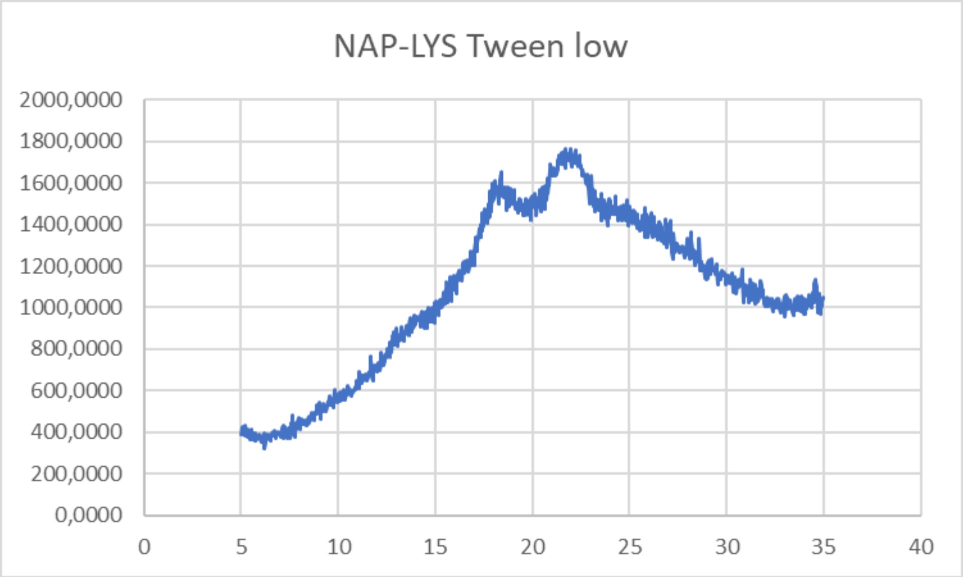
NAP-LYS Tween low
2000,0000
1800,0000
1600,0000
1400,0000
1200,0000
1000,0000
800,0000
600,0000
400,0000
200,0000
0,0000
0
5
10
15
20
25
30
35
40

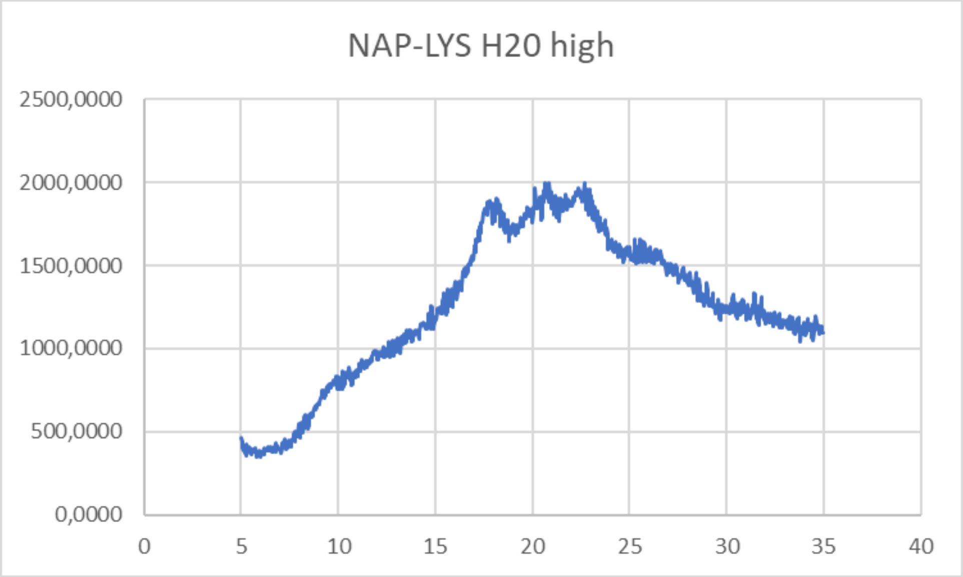

Figure S2. Solid state of freeze-dried concentrations at low and high concentration of detergents

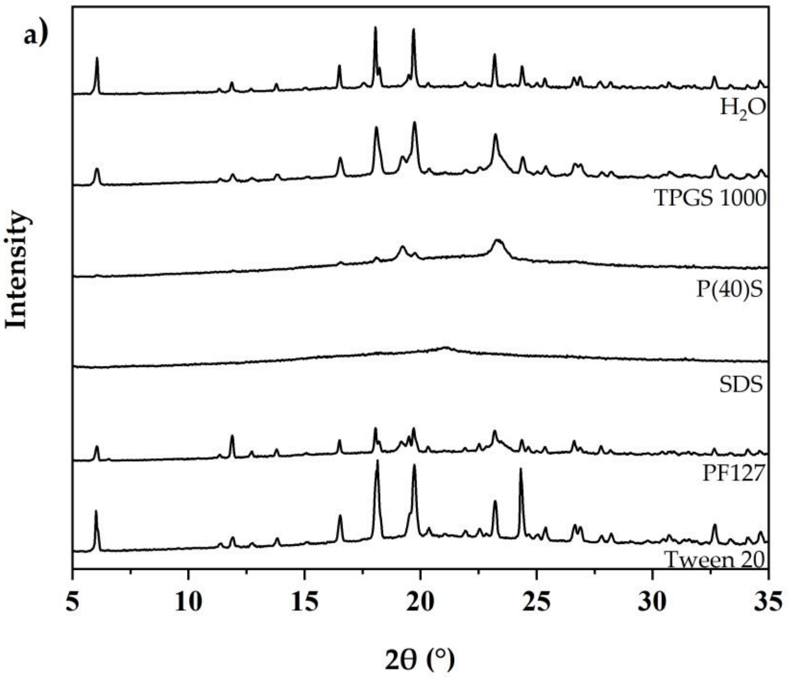
a)
Intensity
H2O
TPGS 1000
P(40)S
SDS
PF127
Tween 20
5
10
15
20
25
30
35
2θ (°)

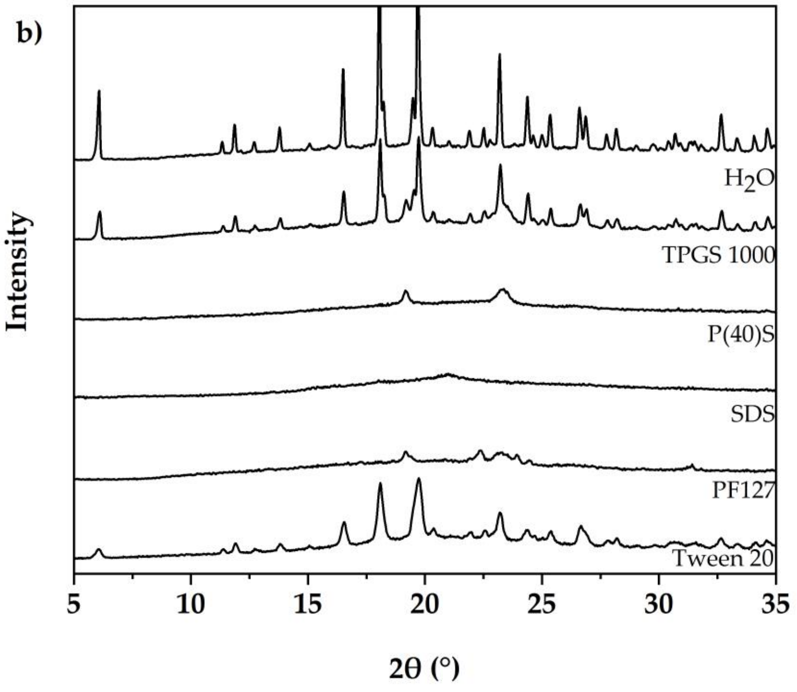
b)
Intensity
H2O
TPGS 1000
P(40)S
SDS
PF127
Tween 20
5
10
15
20
25
30
35
2θ (°)

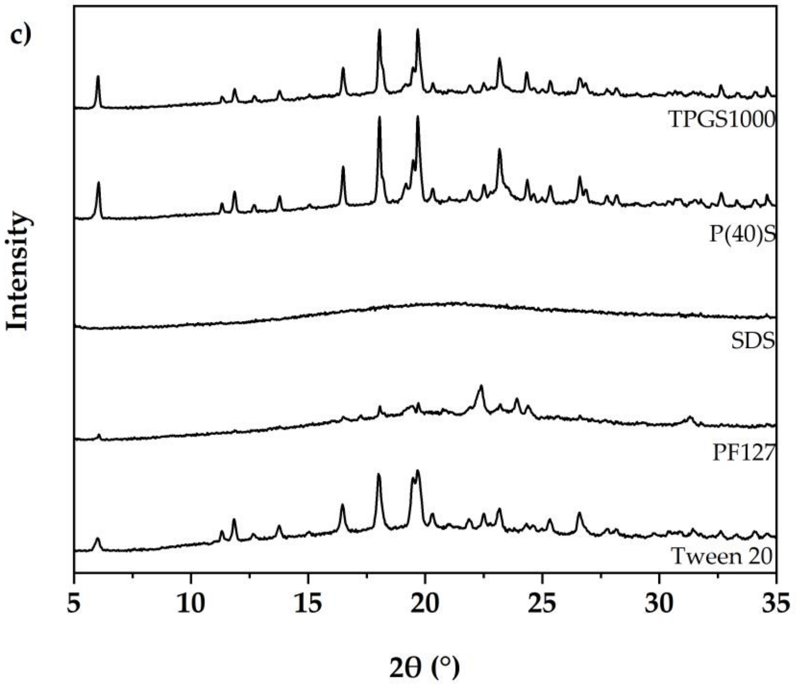
c)
Intensity
TPGS1000
P(40)S
SDS
PF127
Tween 20
5 10 15 20 25 30 35
2θ (°)

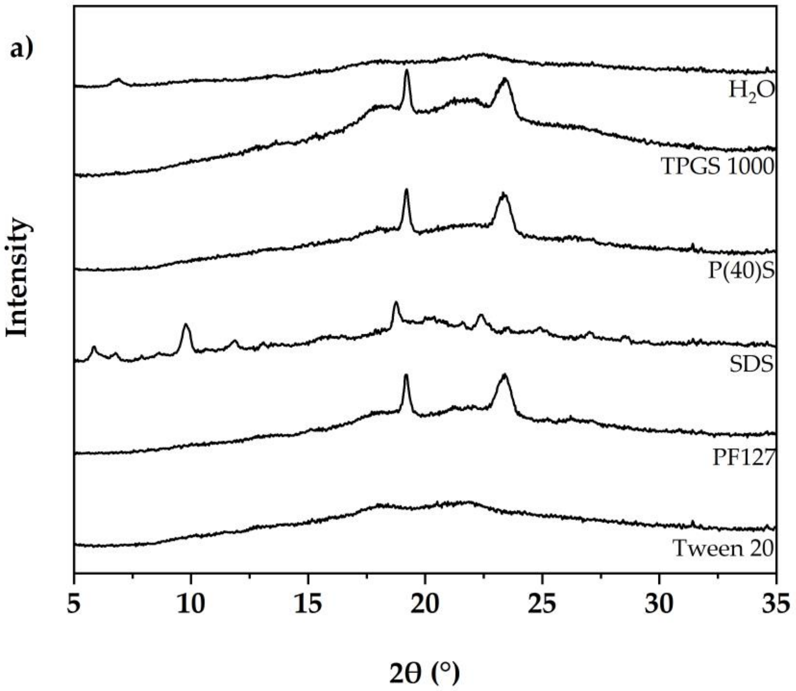
a)
Intensity
H2O
TPGS 1000
P(40)S
SDS
PF127
Tween 20
5 10 15 20 25 30 35
2θ (°)

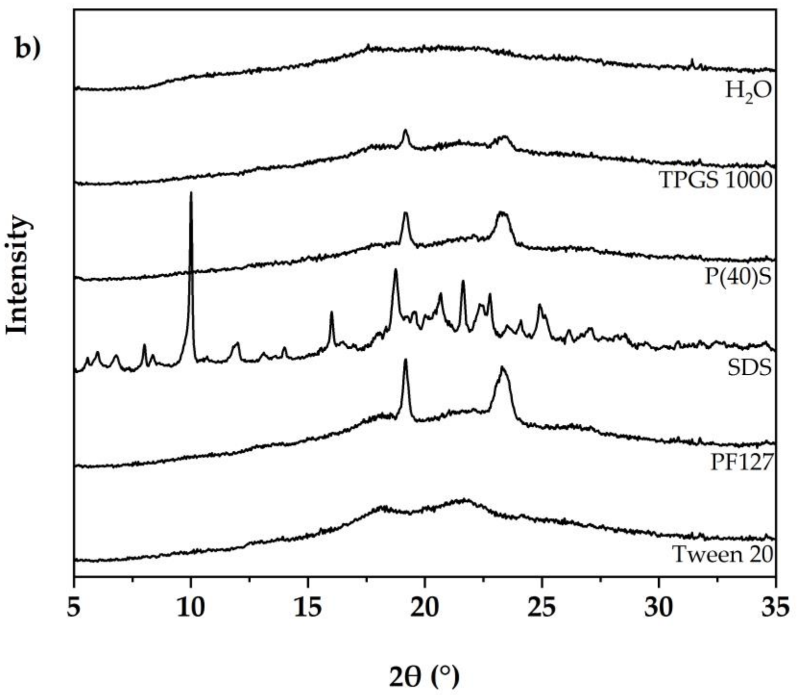

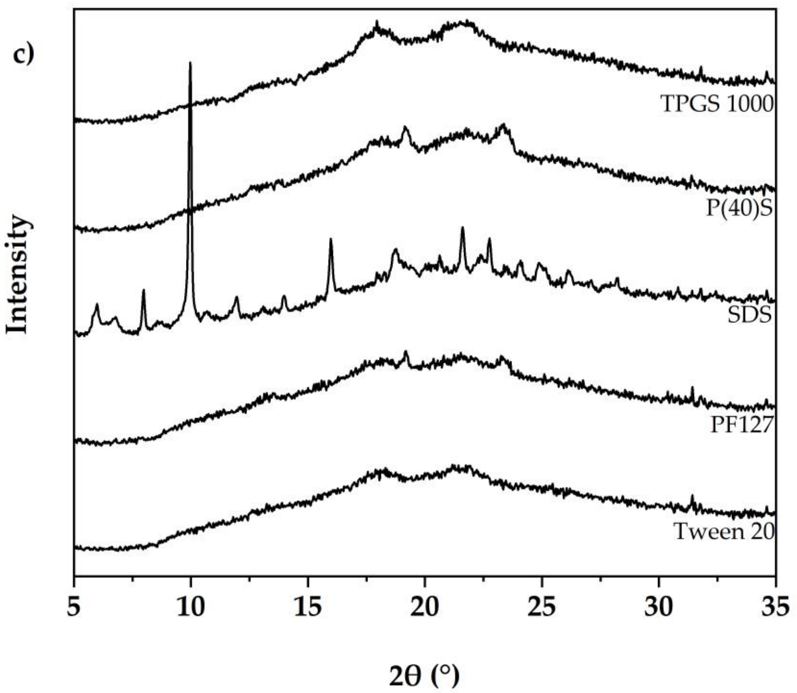

Figure S3. Solid state upon 18 weeks of storage; **a)** NAP-ARG-surfactant high; **b)** NAP-ARG-surfactant medium; **(c)** NAP-ARG-surfactant low. **d)** NAP-LYS-surfactant high; **e)** NAP-LYS-surfactant medium; **f)** NAP-LYS-surfactant low
